# Feature-Specific Event-Related Potential Effects to Action- and Sound-Related Verbs during Visual Word Recognition

**DOI:** 10.3389/fnhum.2016.00637

**Published:** 2016-12-15

**Authors:** Margot Popp, Natalie M. Trumpp, Markus Kiefer

**Affiliations:** Department of Psychiatry, Ulm UniversityUlm, Germany

**Keywords:** grounded cognition, semantics, language, verb processing, event-related potentials

## Abstract

Grounded cognition theories suggest that conceptual representations essentially depend on modality-specific sensory and motor systems. Feature-specific brain activation across different feature types such as action or audition has been intensively investigated in nouns, while feature-specific conceptual category differences in verbs mainly focused on body part specific effects. The present work aimed at assessing whether feature-specific event-related potential (ERP) differences between action and sound concepts, as previously observed in nouns, can also be found within the word class of verbs. In Experiment 1, participants were visually presented with carefully matched sound and action verbs within a lexical decision task, which provides implicit access to word meaning and minimizes strategic access to semantic word features. Experiment 2 tested whether pre-activating the verb concept in a context phase, in which the verb is presented with a related context noun, modulates subsequent feature-specific action vs. sound verb processing within the lexical decision task. In Experiment 1, ERP analyses revealed a differential ERP polarity pattern for action and sound verbs at parietal and central electrodes similar to previous results in nouns. Pre-activation of the meaning of verbs in the preceding context phase in Experiment 2 resulted in a polarity-reversal of feature-specific ERP effects in the lexical decision task compared with Experiment 1. This parallels analogous earlier findings for primed action and sound related nouns. In line with grounded cognitions theories, our ERP study provides evidence for a differential processing of action and sound verbs similar to earlier observation for concrete nouns. Although the localizational value of ERPs must be viewed with caution, our results indicate that the meaning of verbs is linked to different neural circuits depending on conceptual feature relevance.

## Introduction

It is widely accepted that concepts constitute the meaning of words pertaining to different lexical classes such as nouns and verbs (Levelt, 1989; Pulvermüller, 1999; Schomers et al., 2015). Although some models distinguish between general conceptual and lexical semantic systems (Bierwisch and Schreuder, 1992; Paradis, 2004), they all converge on the assumption that concepts are essential for language comprehension and production. Despite the general agreement on the function of conceptual knowledge for object recognition, language and action planning. (Tulving, 1972; Humphreys et al., 1988; Pulvermüller, 1999; Barsalou et al., 2003; Kiefer and Pulvermüller, 2012), it is controversially debated how the conceptual system is functionally organized and implemented in the human brain (for reviews see, Kiefer and Pulvermüller, 2012; Dijkstra and Post, 2015).

In a classical view, conceptual information is represented in an abstract-symbolic format (Collins and Loftus, 1975; Pylyshyn, 1980; Anderson, 1983; Mahon and Caramazza, 2009), supposedly in heteromodal brain areas within anterior (Patterson et al., 2007; Visser et al., 2010) and posterior temporal cortex (de Zubicaray et al., 2001; Gold et al., 2006), which serve as amodal semantic hubs. According to these classical models, the sensory or motor brain systems, only have an accessory role in retrieving conceptual information (Machery, 2007; Mahon and Caramazza, 2008). In support of amodal views of conceptual memory, semantic word processing has been shown to depend on anterior temporal areas (Patterson et al., 2007) as well as on left posterior middle temporal gyrus (pMTG) irrespective of the semantic category or presentation format (Devereux et al., 2013; Fairhall and Caramazza, 2013; Anderson et al., 2015).

In contrast to this classical view, embodiment or grounded cognition approaches (Gallese and Lakoff, 2005; Martin, 2007; Barsalou, 2008; Pulvermüller and Fadiga, 2010; Meteyard et al., 2012; Kiefer and Barsalou, 2013; García and Ibáñez, 2016b) propose that conceptual information is essentially represented in sensory and motor brain areas, depending on the perception and action experience with the environment. Retrieval of conceptual information is thereby instantiated by a partial simulation of previous sensory-motor experiences triggered by language and thought (Barsalou et al., 2003; Kiefer et al., 2008). Evidence favoring grounded cognition theories mainly comes from behavioral, neuroimaging, and neuropsychological studies demonstrating a differential involvement of the sensory and motor systems depending on the conceptual and/or lexical category (Barsalou, 2008; Pulvermüller and Fadiga, 2010; Kiefer and Pulvermüller, 2012; Kiefer and Barsalou, 2013; Moseley et al., 2016). Several studies compared the processing of nouns vs. verbs (Perani et al., 1999). It has been suggested that the meaning of nouns predominately relates to visual object information (Jones and Smith, 1993; Setti et al., 2009), whereas the meaning of verbs is mostly characterized by action information (Glenberg and Gallese, 2012; Moseley and Pulvermüller, 2014). Correspondingly, processing of nouns was associated with increased activity within visual brain areas. Verbs, in contrast, elicited more cortical activity within the motor system (Pulvermüller et al., 1999a). The claim that differences in word processing may be due to different word classes (for a review see, Vigliocco et al., 2011), but not to different semantic features (Shapiro and Caramazza, 2003; Bedny et al., 2008; Mahon and Caramazza, 2008) is predominately supported by neurophysiological (Damasio and Tranel, 1993; Daniele et al., 1994) but also by behavioral (Pechmann et al., 2004; Vigliocco et al., 2004), electrophysiological (Preissl et al., 1995; Barber et al., 2010), or imaging studies (Perani et al., 1999; Shapiro et al., 2006). However, many of these studies lack in evaluating the semantic content of object-related and action-related information of nouns vs. verbs and are therefore more likely to be interpreted as an object-action association (Vigliocco et al., 2011). In contrast, one study indicated that the differential processing of verbs compared to nouns cannot be reduced to word class differences (Moseley and Pulvermüller, 2014).

However, processing differences between verbs and nouns may not only be caused by grammatical categories or conceptual feature types. Compared to nouns, verbs implicate a more complex processing as they are more likely to have multiple meanings (Ehrlich and Rayner, 1981; Zola, 1984). This is linked to more complex syntactic subcategorizations and argument structures (Shapiro et al., 1989) and results in slower reaction times for verbs than for nouns (Kauschke and Stenneken, 2008; Palazova et al., 2011). For verbs being more difficult in processing than nouns, their age of acquisition is delayed (Gentner, 1982). Verbs were also found to be less imageable and more abstract than nouns (Bird et al., 2001; Colombo and Burani, 2002). These factors can therefore reduce the comparability of verbs and nouns.

In order to avoid these difficulties associated with word class differences, a promising way to assess the structure of the semantic system is to investigate semantic word category effects within a given word class. Within the domain of nouns, such word category effects have been intensively investigated: Action-related tool concepts activated motor areas in frontal and parietal motor cortex as well as motion-related areas in posterior temporal cortex (Chao et al., 1999; Chao and Martin, 2000) while sound-related nouns activated auditory brain areas in superior and middle temporal cortex (Kiefer et al., 2008, 2012). Focal damage to this auditory association area in a brain-lesioned patient specifically impaired the retrieval of sound-related concepts (Trumpp et al., 2013a). Nouns related to visual conceptual information, in contrast, activated visual regions in occipito-temporal cortex (Chao and Martin, 1999). However, category-specific deficits in brain-damaged patients are not necessarily linked to deficits in conceptual knowledge of objects (Capitani et al., 2003).

Several event-related potential (ERP) studies tracked the time course of semantic noun processing and compared ERP activity related to action-related vs. sound-related words: Action-related nouns elicited relatively more positive ERPs at fronto-central electrodes whereas sound-related nouns were associated with more negative ERPs over this scalp region (Kiefer et al., 2008; Trumpp et al., 2013b, 2014). These ERP effects emerged at about 200 ms suggesting that they reflect early access to conceptual feature rather than late post-conceptual imagery. Furthermore, pre-exposure of the critical words within a priming paradigm, leads to a specific modulation of these feature-specific ERP effects. Priming sound nouns reduced the negative fronto-central ERP shift whereas priming action nouns attenuated the positive fronto-central ERP shift. Source analyses for these feature-specific ERP priming effects suggested generators within and close to frontal and parietal motor areas for action words, and auditory association areas in temporal cortex for sound words (Trumpp et al., 2013b, 2014).

Unlike in nouns, feature-specific conceptual category differences in verbs mainly focused on action concepts and assessed body part specific effects. Body part related verbs (e.g., pick, lick, or kick) were found to somatotopically activate the same motor and premotor brain areas that are also active when performing real actions with hand, tongue, or leg, respectively (Hauk et al., 2004). A functional connection between these different categories of action verbs and the cerebral motor system was demonstrated by action interference experiments (Shebani and Pulvermüller, 2013) and transcranial magnetic stimulation (TMS) studies (Pulvermüller et al., 2005) showing effector-specific effects. However, some studies failed to replicate a somatotopic organization of effector-specific action word representations in motor areas (Arévalo et al., 2007; Postle et al., 2008; for review see, Cardona et al., 2013). The functional significance of motor areas for action word processing has been also demonstrated in patients with neurodegenerative motor diseases, in which specific impairments in action verb processing was found (Bak, 2013; García and Ibáñez, 2014; Bocanegra et al., 2015). Recent theories thus propose a functional connection between language development and specific neuronal motor control (Glenberg and Gallese, 2012) and suggest a tight coupling of action and language such as the Hand-Action-Network Dynamic Language Embodiment model (García and Ibáñez, 2016b). However, feature-specific conceptual category differences such as between action- vs. sound-related words have not been previously investigated in verbs.

Using ERP recordings, the present work aimed at assessing whether feature-specific ERP differences between action and sound concepts, as previously observed in nouns (Trumpp et al., 2013b, 2014), can be found within the word class of verbs. Furthermore, as effector-specific activation in action verbs has not always been replicated (Postle et al., 2008; Arévalo et al., 2012) the assessment of category effects across feature types such as action vs. sound would be a promising novel approach to test grounded cognition theories in the domain of verbs. In Experiment 1, participants were visually presented with matched sound and action verbs within a lexical decision task, which provides implicit access to word meaning and minimizes strategic access to semantic word features. Experiment 2 tested whether pre-activating the verb concept in a context phase, in which the verb is presented with a related context noun, modulates subsequent feature-specific action vs. sound verb processing within the lexical decision task. A modulation of feature-specific ERP effect by pre-activating the verb concept in a context phase would further substantiate the semantic nature of the ERP effects for action and sound verbs. We assumed that presenting the words in a context before the critical lexical decision task would prime the concept in modality-specific areas, thereby specifically deactivating corresponding modality-specific cortex depending on the conceptual feature type. Such an electrophysiological dissociation between action and sound verbs across experiments would point to a modality-specific representation of conceptual action and sound features also for verbs substantiating the generality of grounded cognition approaches across word classes.

## Experiment 1

In Experiment 1, action and sound verbs were presented together with control words in a lexical decision task. Control verbs had no specific relevance of action or sound features. We investigated whether action and sound verbs would elicit differential ERP effects compared with control words: Based on previous findings obtained with nouns, we expected that verb category ERP effects should emerge with an onset of about 200 ms. Action verbs should be associated with a more positive potential at fronto-central electrodes compared with control and sound words. Sound verbs in contrast were expected to elicit a more negative potential compared with control and action verbs.

### Materials and methods

#### Participants

Twenty-four (mean age: 23.6 years, range: 20–27 years, 13 female) right-handed (Oldfield, 1971) healthy volunteers participated in Experiment 1. Two subjects were excluded from analyses due to slow lexical decision RTs deviating 2 *SD*s from the sample mean. In both experiments, subjects were native German speakers with normal or corrected to normal vision and no history of psychiatric or neurological disorders. Subjects gave written informed consent and were paid 17 Euro for participation. The procedures were approved by the local Ethical Committee.

#### Stimuli

569 German verbs in their infinitive form were rated according to specified conceptual features which were assigned to 30 volunteers not participating in the main experiment. The verbs had to be rated with respect to their relevance of visual, action, sound, and emotional features (“How strong do you associate the named verb with actions, sounds, visual features, or emotions?”) as well as their familiarity (“How familiar is the word to you?”) on a six-point Likert scale (one = low relevance/familiarity; six = high relevance/familiarity). The verb set was equally split into two lists, in order to reduce the number of verbs to be rated by each individual participant. Furthermore, ratings of visual, action, and sound features were obtained in questionnaires different from ratings of emotions and familiarity, in order to reduce the length of the questionnaires. Hence, there were four questionnaires in total, which were assigned to 15 volunteers each, who were different from the main experiment.

In order to create stimulus lists for action and sound words, non-ambiguous words with a relevance rating of four or higher for the critical features action or sound were selected. Action or sound words with a high rating for any other feature (>4.5) were excluded from word lists. An additional list of control words with low feature relevance (<4.0) for both action and sound was also created. The final word lists of 40 action verbs, 40 sound verbs, and 40 control verbs were matched for familiarity, emotionality, relevance of visual features, word length, word frequency, lemma frequency, type frequency, bigram frequency, and trigram frequency (see Table 1). Word frequency was determined by using the CELEX database (Baayen et al., 1995). All other psycholinguistic word features were determined by using the dlex database (Heister et al., 2011). Action verbs and sound verbs differed from control verbs with regard to action [*p* < 0.0001; t_(78)_ = 20.47] and sound features [*p* < 0.0001; t_(78)_ = 24.68], respectively, but were comparable to all other conceptual and linguistic measures (all *p* > 0.05). Action verbs had significantly higher ratings [*p* < 0.0001; t_(78)_ = −14.77] for the relevance of action features and significantly lower relevance [*p* < 0.0001; t_(78)_ = 24.07] of sound features than sound verbs. Action and sound verbs did not differ from each other in most of the other measures (all *p* > 0.05). However, action verbs had a slight, but significantly higher relevance of visual features than sound verbs [*p* < 0.008; t_(78)_ = −2.72], presumably due to the importance of visual features for action execution (Johansson et al., 2001) making a perfect match difficult.

**Table 1:** **Matching of conceptual and psycholinguistic stimulus features for critical action and sound verbs of Experiment 1 and 2 as well as for control verbs of Experiment 1**.

	**Action**	**Acoustic**	**Visual**	**Familiarity**	**Emotion**	**Word length**	**Word frequency**	**Lemma. frequency p.Mio.**	**Character. bigram. frequency p.Mio.**	**Character. trigram. frequency p.Mio.**
Action verbs	5.14	1.93	2.74	4.00	2.64	7.28	194.98	32.97	996151.77	486060.42
Sound verbs	3.03	4.97	2.32	3.65	2.78	7.30	145.93	23.39	957488.78	468888.67
Control verbs	3.12	1.80	2.49	3.89	2.88	7.35	131.00	28.42	981487.94	456490.97
Action vs. sound verbs (*p*-values)	<0.0001	<0.0001	0.008	0.06	0.43	0.94	0.66	0.58	0.55	0.46
Action vs. control verbs (*p*-values)	<0.0001	0.36	0.06	0.58	0.14	0.84	0.41	0.76	0.84	0.20
Sound vs. control verbs (*p*-values)	0.55	<0.0001	0.33	0.21	0.52	0.88	0.88	0.75	0.72	0.63

*Depicted are p-values of two-tailed t-tests*.

Pseudowords were derived from verbs, which were not used for the experimental word lists, by replacing one consonant and one vowel by another consonant and vowel resulting in pronounceable, but meaningless letter strings. Pseudowords were matched in word length for all experimental verb lists (all *p* > 0.05).

#### Procedure

40 action verbs (to throw), 40 sound verbs (to crackle), 40 control verbs (to avoid) and 120 pseudoverbs (schliken) were presented in random order in white font (16 point character height) against a black background on a CRT computer screen synchronous with the screen refresh (refresh rate 16 ms). At a distance of 70 cm the viewing angle for the stimuli subtended about 3° horizontally and 1° vertically. Each trial started with a fixation cross in the middle of the screen, which was presented for 500 ms, followed by the presentation of the verb or the pseudoverb for 400 ms. Participants were instructed to press the left button on an external keyboard with their right index finger in response to a word and to press the right button with their right middle finger in response to a pseudoword. Instructions stressed both speed and accuracy of the responses. The screen remained blank until the response was given and for 500 ms thereafter. Three hash marks indicated a pause between the trials and lasted 1500 ms on average. Pause times (1000, 1250, 1500, 1750, and 2000 ms) were distributed equally across each condition. Stimulus delivery and response collection was controlled by Experimental Runtime System software (BeriSoft Coorperation, Frankfurt, Germany). Prior to each session, participants were able to practice the task with 12 stimuli not used for the main experiments and repeated the training if necessary. The experimental session lasted about 20 min.

#### EEG-recording, signal extraction, and statistical analysis

Participants were comfortably seated in an upright position in a dimly illuminated, sound attenuated, and electrically shielded cabin. Participants received detailed written and verbal instructions and they had to practice the task to ensure that they completely understood the instruction. They were also told to be relaxed during the electroencephalography (EEG)-recordings and to only blink during the breaks.

Forty-six equally distributed sintered Ag/AgCl elektrodes were placed on the participants' heads with an elastic textile cap (EasyCap, Herrsching, Germany). The reference electrode was positioned between FCz and Cz, and the ground electrode between the AFz and Fz. The electrodes' impedance was kept below 5 kΩ. Eye movements were monitored with supra- and infra-orbital electrodes and with electrodes on the external canthi. Scalp potentials were continuously recorded (low-pass filter: 70 Hz, 24 dB/octave attenuation, 50 Hz notch filter) using BrainAmp amplifiers (BrainProducts, Gilching, Germany) and digitized at a sampling rate of 500 Hz.

EEG data were processed by BrainVision Analyzer 2.0 (BrainProducts, Gilching, Germany). Raw data were digitally filtered (high-pass: 0.1 Hz, 12 dB/octave; low pass: 30 Hz, 24 dB/octave). Ocular artifacts were removed by independent component analysis (Makeig et al., 1997). Data of single noisy electrodes were replaced by interpolated data of 4 surrounding electrodes by Hjorth Nearest Neighbors interpolation. Continuous EEG was segmented from 150 ms prior to 1000 ms after the target, and baseline corrected (−150 to 0 ms). Artifact-free EEG segments of trials with correct responses were averaged separately for each condition in each participant to extract ERPs. Individual ERPs were re-referenced to average reference (Bertrand et al., 1985; Kiefer et al., 1998). We analyzed electrodes in scalp regions of interest because we had apriori predictions regarding the topography of the feature-specific effects based on findings from earlier studies with nouns (Trumpp et al., 2013b). Similar to previous studies with nouns (Trumpp et al., 2013b), ERPs were statistically analyzed at contralateral pairs of electrodes in a fronto-central scalp region (C3/C4, FC1/FC2, CP1/CP2). As visual inspection of the data revealed a somewhat more posterior distribution of the fronto-central feature-effect compared with the study on nouns (AF3/AF4, F1/F2, and FC1/FC2), the set of analyzed electrodes overlaps, but is not identical. Furthermore, as the ERP effects in the present study extended to parietal electrodes, electrodes in this scalp region were also analyzed (PO3/PO4, P1/P2, CP3/CP4). These differences compared to the study in nouns may reflect interindividual variation in brain anatomy of the different samples or the use of material from different lexical classes (see also the General Discussion section). For each scalp region, repeated-measures analyses of variance (ANOVA) were performed on mean voltages within three time windows: 180–280, 280–380, and 380–480 ms similar to earlier studies (Kiefer et al., 2008; Trumpp et al., 2013b, 2014). In a first step, the analyses of variance (ANOVAs) included the factors scalp region (centro-parietal, fronto-central), feature type (sound, action, control words), hemisphere (left, right), and electrode site. Subsequent analyses were performed separately for each scalp region and included the factors feature type, hemisphere and electrode site. Significant effects were further assessed using Tukey's HSD *post-hoc* tests. The mean number of artifact-free EEG segments of trials with corrects responses was 36.41 (*SD* = 2.42) for sound verbs, 38.27 (*SD* = 1.24) for action verbs, and 37.41 (*SD* = 2.13) for control verbs. Although the difference was small, it significantly differed across conditions [*F*_(2, 42)_ = 7.75; *p* = 0.0014]. *Post-hoc* test revealed that this effect is based on a significant difference between action and sound verbs (*p* = 0.0009). Control verbs did not differ from the other feature categories.

In addition to the parametric statistical analyses in selected scalp regions and time windows, we performed non-parametric cluster permutation tests across all electrode sites and time points using BESA Statistics 2.0 [BESA GmbH, Graefelfing, Germany, (Maris and Oostenveld, 2007)], in order to confirm the robustness of our findings with an entirely data-driven statistical test. In preparation for permutation testing, ERP data were first analyzed with repeated-measures univariate ANOVAs with the factor feature type (sound, action, and control) to identify differences between categories: Running *F*-tests were calculated for all time points (−150 ms prior stimulus presentation until 700 ms), which were then used for following permutation testing. Analyses focused on effects prior to 700 ms after stimulus presentation in order to exclude post-lexical semantic processes. Pair-wise *post-hoc* permutations tests were calculated using Scheffé tests, in order to identify clusters, in which ERPs between the three conditions significantly differ. Permutation testing was corrected for multiple comparisons by combining the building of a cluster value with permutation testing. Statistical testing was based on 1000 permutations. Clusters across electrodes and time points were identified as significant, when their cumulated statistical cluster values were higher than 99.8% of all clusters derived by random permutation of data. Results therefore can be considered corrected for multiple comparisons.

### Results

#### Behavioral results

Mean accuracy of the lexical decision task was 94% (*SD* = 3.3%). This shows that participants performed the task carefully. An ANOVA revealed that number of errors differed between sound verbs (3.05), action verbs (1.23), and control verbs (2.05) [*F*_(2, 42)_ = 12.06; *p* < 0.0001; $\eta_{p}^{2}$ = 0.36]. *Post-hoc* tests revealed larger error rates for sound verbs than for action (*p* = 0.0002) and control verbs (*p* = 0.03). Mean error rates for action and control verbs did not significantly differ. For reaction time (RT) analysis, mean RT of correct responses was calculated for each word category. Outlying data (mean +/− 2 *SD*) were rejected from analysis (4.21%). Mean reaction times did not significantly differ between action verbs (592 ms), sound verbs (594 ms), and control verbs (587 ms) [*F*_(2, 42)_ = 1.07; *p* = 0.35]. In order to test, whether response speed or error rate in the lexical decision task unspecific ally affected ERPs, several control analyses were performed by dividing the participants in fast vs. slow responders and in high vs. low error rate groups, respectively, based on a median split (see below).

#### Electrophysiological results

**Figure 1:**
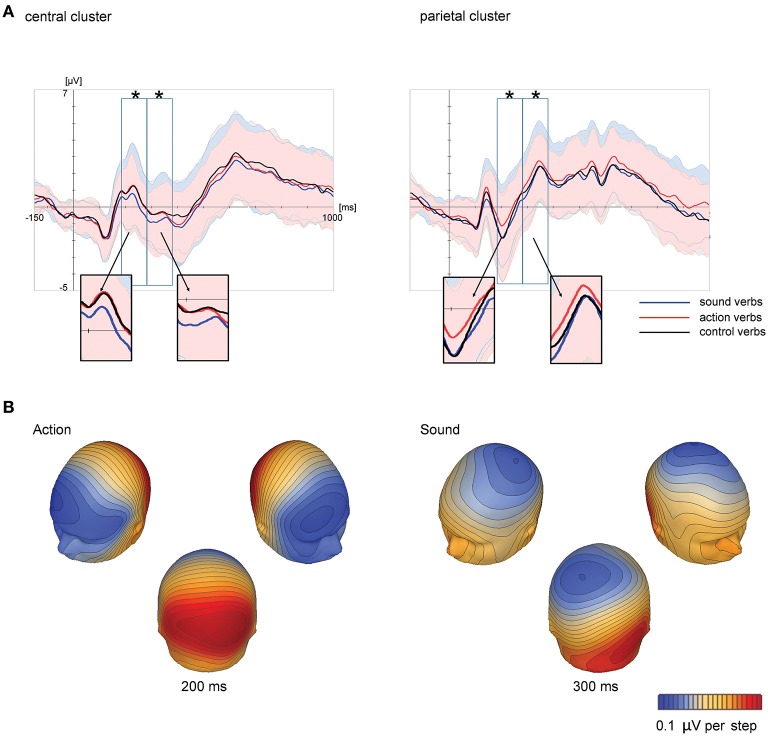
**Grand-averaged ERPs at central and parietal scalp regions, averaged across electrode sites within each scalp region, in Experiment 1 (A)** as a function of feature type (action, sound, and control verbs). Time windows analyzed statistically (180–280, 280–380, and 380–480 ms) are marked by black rectangles. Colored shadings indicate standard deviations. Significant ERP effects of feature type are highlighted by asterisks. Corresponding topographical potential maps at the time point of maximum global field power are shown **(B)** for ERP differences between action and control verbs as well as been sound and control verbs.

**Figure 2:**
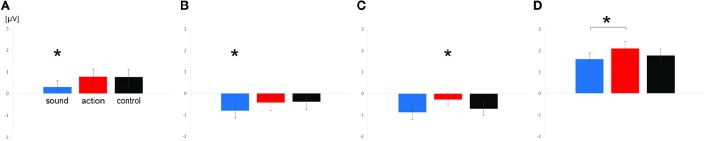
**Means and standard errors of analyzed scalp regions in Experiment 1**. Depicted are mean ERPs of sound, action, and control verbs over central scalp regions for the time window 180–280 ms **(A)** and 280–380 ms **(B)** and over parietal scalp regions for the time window 180–280 ms **(C)** and 280–380 ms **(D)**. Significant effects are highlighted by asterisks.

At the central scalp region, grand averaged ERPs suggested a relatively more negative scalp potential for sound verbs than for action verbs and control verbs starting at 180 ms. Action verbs, however, elicited a relatively more positive scalp potential than sound verbs and control verbs over the parietal scalp region in a similar time range (see Figure 1A). These feature-specific effects were further assessed by statistical analyses. In order to reduce the complexity of results, we only report effects involving the factor feature type. ERP means and standard errors are depicted in Figure 2.

##### Time window 180–280 ms

The first ANOVA including the factors scalp region, feature type, hemisphere and electrode site revealed a significant interaction of the factors feature type and scalp region [*F*(_1, 21)_ = 7.97; *p* = 0.01; $\eta_{p}^{2}$ = 0.15]. Subsequent analyses within each scalp region revealed significant main effects for the factor feature type over both the central [*F*_(2, 42)_ = 4.81; *p* = 0.01; $\eta_{p}^{2}$ = 0.19) and parietal scalp region [*F*_(2, 42)_ = 7.71; *p* = 0.001; $\eta_{p}^{2}$ = 0.27]. At central electrodes, *post-hoc* tests showed that this effect was based on significant ERP differences between sound verbs compared to action (*p* = 0.025) and control verbs (*p* = 0.03). The latter did not differ from each other. Sound verbs elicited a relatively more negative scalp potential than action verbs and control verbs. Over the parietal scalp region, *post-hoc* tests revealed significant scalp potential differences between action verbs compared to sound (*p* = 0.001) and control verbs (*p* = 0.024), the latter did not differ from each other. Over the parietal scalp region, action verbs elicited a relatively more positive scalp potential than sound and control verbs.

##### Time window 280–380 ms

The first ANOVA including the factor scalp region only yielded a main effect of feature type [*F*_(2, 42)_ = 5.43; *p* = 0.008; $\eta_{p}^{2}$ = 0.21] and a main effect for scalp region [*F*_(1, 21)_ = 44.23; *p* < 0.0001; $\eta_{p}^{2}$ = 0.68], but only a tendency toward an interaction (*p* = 0.12). Separate ANOVAs for each scalp region confirmed that the effect of feature type was significant over both the central [*F*_(2, 42)_ = 5.64; *p* = 0.007; $\eta_{p}^{2}$ = 0.2] and parietal scalp region [*F*_(2, 42)_ = 3.76; *p* = 0.031; $\eta_{p}^{2}$ = 0.15]. At central electrodes, ERPs of sound verbs differed from those to action (*p* = 0.02) and control verbs (*p* = 0.01) according to *post-hoc* tests. ERPs of action and control verbs were not significantly different. Sound verbs elicited a relatively more negative scalp potential than action and control verbs. At parietal electrodes, ERP differences between action and sound verbs were statistically reliable (*p* = 0.027), but control verbs did not differ for either condition. As in the previous time window, action verbs elicited a more positive scalp potential than sound verbs, while the scalp potentials of control verbs were in between.

##### Time window 380–480 ms

ANOVA including the factor scalp region revealed a main effect of scalp region [*F*_(1, 21)_ = 64.45; *p* < 0.0001; $\eta_{p}^{2}$ = 0.75] but no significant interaction with the factor feature type (*p* = 0.33). Separate ANOVAs for each scalp region did not reveal any significant main effects or interactions neither at the central nor at the parietal scalp region.

##### Control analyses with regard to response speed and errors

In order to assess, whether feature-specific ERP effects would depend on response speed, we analyzed the relationship between ERPs and RT by dividing the participants of our sample in fast and slow responders based on a median split and included the factor response speed as additional factor in the ANOVAs. The ANOVA with between-subject factor response speed revealed no significant interactions with the factor feature type (all *p* > 0.18) indicating that feature effects were not compromised by RT differences. We also analyzed the relationship between ERPs and error rate by dividing the participants in low and high error rate groups by a median split including the factor error rate group as additional factor in the ANOVAs. These analyses only revealed a significant interaction between feature type and error rate group over the central scalp region in the time window of 280–380 ms [*F*_(2, 40)_ = 3.43, *p* = 0.042, $\eta_{p}^{2}$ = 0.15]. The effect of feature type was in the same direction in both error rate groups, but was more pronounced in the participant group with a high error rate. In the other time windows and scalp regions the interaction between feature type and error rate group was not significant (all *p* > 0.15). Overall, these control analyses render it unlikely that response speed or error rate had a systematic influence on the observed effects of feature type.

##### Non-parametric cluster permutation tests

**Figure 3:**
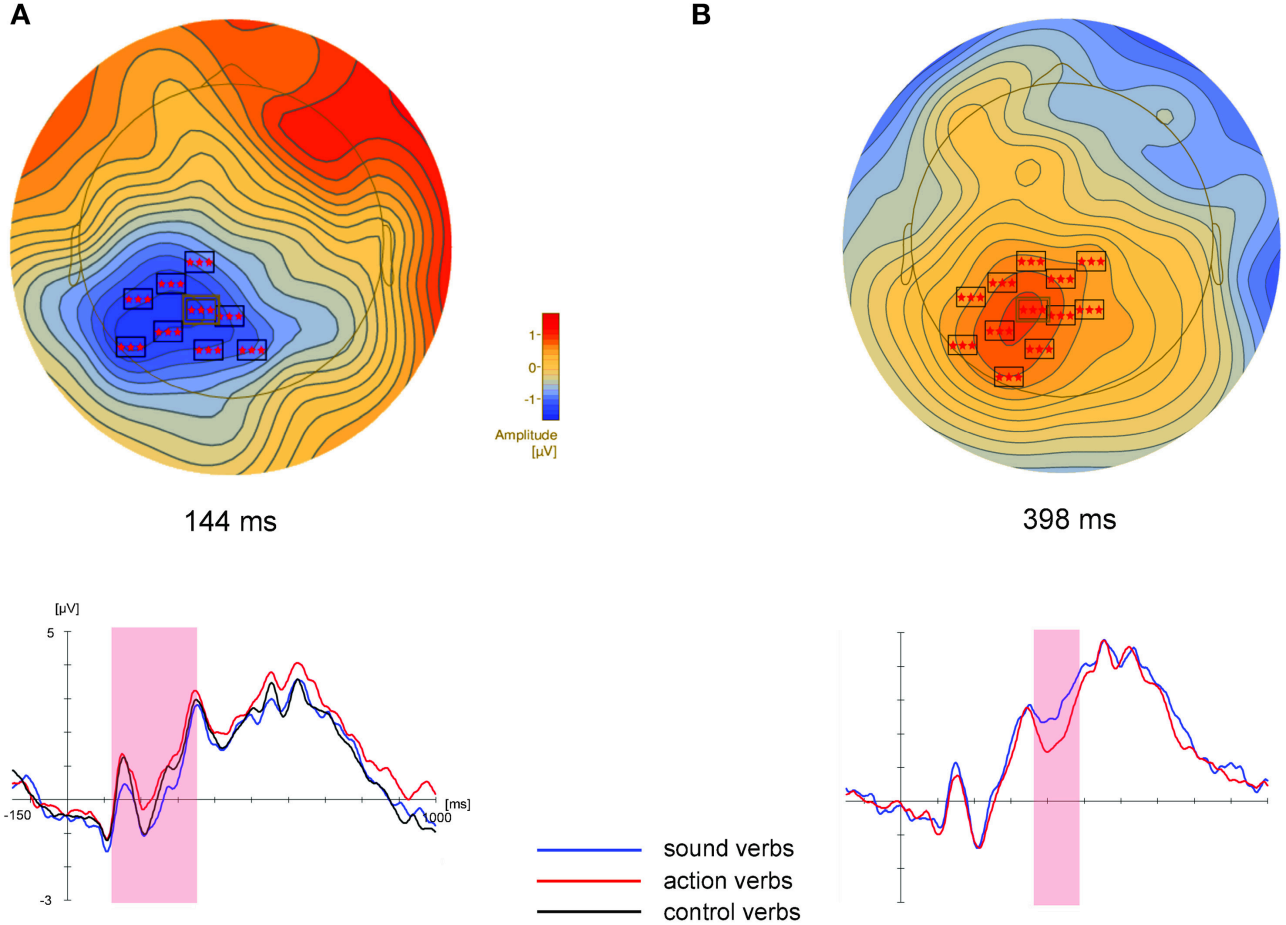
**Results of the cluster permutation tests for the sound vs. action feature comparison**. Above: Topographic map of the large centro-parietal cluster at the time point of the highest *F*-value across all electrodes of this cluster (electrode P1, highlighted in the map). Shown are only electrodes of this cluster with significant *F*-values (*p* < 0.05) at the depicted time point. For all electrodes within clusters and all clusters, we refer to Tables 2, 3. Cluster electrodes are overlaid on interpolated potential differences between sound and action conditions. Below: Grand-averaged ERPs for peak electrode P1 of these clusters. Colored shadings indicate the significant time window of the corresponding cluster. **(A)**: Experiment 1, **(B)**: Experiment 2.

Cluster permutation tests revealed differences between sound and action verbs in a cluster of centro-parietal electrodes between 110 and 346 ms (Table 2, Figure 3A), where sound verbs elicited a more negative potential than action verbs. This feature effect in the centro-parietal cluster was accompanied by a polarity-reversed effect in a fronto-temporal electrode cluster (114–158, 188–272 ms). Such concurrent bipolar voltage patterns are typical in average-referenced data sets and most likely reflect activity of the same underlying set of neural generators (see Figure 1). A significant difference between sound vs. control verbs was found in a central electrode cluster in the time window from 232 to 338 ms, where sound verbs elicited a more negative scalp potential than control verbs. For the comparison action vs. control verbs a significant parietal electrode cluster was obtained in the time window from 112 to 232 ms, in which action verbs elicited a more positive scalp potential than control verbs. Data-driven non-parametric cluster permutation tests thus yielded quite comparable results as the parametric statistical analyses of selected scalp regions and time windows.

**Table 2:** **Results of cluster permutation tests for Experiment 1**.

	**Electrodes within cluster**	**Time window**	***p*-value**
Sound vs. action	P7, C5, P5, PO3, PO1, P1, CP3, C3, FC1, CP1, Cz, P6, PO4, PO2, Pz, P2, CP4, C4, CP2, CPz	110–346 ms	<0.00001
	F10, FT8, AF8, FP2, AF4, F9, FC6	114–158 ms	0.002
	F9, FT9, T7, FT7, AF7	188–272 ms	0.001
	P7, TP7, P5, PO3, PO1, P1, CP3, C3, CP1, Pz, CPz	508–612 ms	0.001
	P9, P7, TP7, C5, P5, PO3, P1, CP3, CP1	620–676 ms	0.001
Sound vs. control	O9, O1, P7, TP10, P10, O10, Iz, Oz, O2, P8, TP8, T8, PO4	130–196 ms	0.001
	PO3, P1, CP3, C3, FC1, CP1, Pz, CPz	134–178 ms	0.001
	CP3, C3, FC1, CP1, Cz, FC2, CP2, CPz	232–338 ms	0.001
	TP9, P9, O9, TP10, P10, O10, Iz	302–344 ms	0.001
	CP1, Cz, CP4, C4, FC2, CP2, CPz	378–412 ms	0.001
	CP3, C3, FC1, CP1, Cz, FC2, CP2, CPz	502–570 ms	0.001
	TP10, P10, O10, TP8	510 – 636 ms	<0.00001
	FPz, AFz, AF3, F5, F1, Fz, FCz, AF4, F2, FC2	580–654 ms	0.001
Action vs. control	O9, O1, P7, P5, PO3, PO1, P1, CP3, TP10, P10, O10, Iz, Oz, O2, P8, TP8, P6, PO4, PO2, P2	112–232 ms	0.001
	FT7, AF7, FP1, FPz, AFz, AF3, F5, Fz, AF8, FP2, AF4, F9, FC6, FC4, F2	170–238 ms	0.001
	TP9, P9, O9, O1, P7, TP7, P5, PO3, TP10, P10, O10, Iz, O2, O2	364–430 ms	0.001
	AF7, FP1, FPz, AFz, AF3, F1, Fz, FCz, FT8, AF8, FP2, AF4, F9, FC6, FC4, F2, FC2	520–800 ms	0.001
	TP9, P9, O9, P7, TP7, P5, PO3, PO1, P1, TP10, P10, O10, Oz, O2	520–590 ms	0.001
	TP9, P9, O9, O1, P7, TP7, C5, P5, PO3, PO1, P1, CP3, TP10, P10, O10, Iz, Oz, O2, P8, TP8	582–762 ms	<0.00001

### Discussion

Experiment 1 revealed differential feature-specific ERP effects of action and sound verbs compared to control verbs: While action verbs elicited a significantly more positive scalp potential at the parietal scalp region, sound verbs elicited a significantly more negative scalp potential at a central scalp region. Both feature-specific effects started at about 150 ms after stimulus presentation. Parametric analyses of ERPs in selected time windows and scalp regions of interest were confirmed by data-driven analysis with non-parametric cluster permutation tests.

As the feature-specific effects had an early onset, they most likely reflect rapid access to conceptual features rather than strategic imagery effects, which are known to modulate late ERPs (Nittono et al., 2002; Swaab et al., 2002; Barber et al., 2013). The time course, the topography and the polarity of the ERP differences between sound and action verbs were largely comparable to previous studies with nouns (Kiefer et al., 2008; Trumpp et al., 2013b). However, in contrast to the findings with nouns, the present feature-specific ERP effects of verbs extended to parietal electrodes. Since different samples are involved, it is difficult to draw safe conclusion whether this more parietal effect for verbs simply reflects slight interindividual differences in neuroanatomy or indexes word class differences in the representation of action information. Furthermore, the more parietal distribution of the feature-effect might have its origin that action and sound verbs could not be perfectly matched for visual conceptual context. This issue is further elaborated in the general discussion section. Apart from this difference compared with earlier study on nouns, our results show that feature-specific effects for action and sound can also be obtained by using verbs.

## Experiment 2

Experiment 2 tested whether pre-activating the verb concept in a context phase, in which the verb is presented with a related context noun (for example: Ball–to throw), modulates subsequent feature-specific action vs. sound verb processing within the lexical decision task. A modulation of feature-specific ERP effects by pre-activating the verb concept in a context phase would further substantiate the semantic nature of the ERP effects for action and sound verbs. Differential neurophysiological repetition effects as a function of stimulus categories have been a valuable and highly sensitive tool in the past to identify the nature of underlying stimulus representations (Henson, 2003; Kiefer, 2005).

Experiment 2 comprises two phases: In the context phase, subjects had to decide whether or not a context noun is semantically related to a verb. Critical stimuli were always presented in semantically related trials. In the second phase, verbs were presented within a lexical decision task as in Experiment 1. In Experiment 2, the control words were omitted, because pilot studies indicated that it was difficult to create a matching context condition for the control verbs. We assumed that processing the verb together with a context word in the context phase pre-activates its meaning leading to deactivation of the corresponding feature representation in the subsequent lexical decision task. Similar to feature-specific ERP repetition priming effects in nouns (Kiefer, 2005; Sim and Kiefer, 2005; Trumpp et al., 2014), we expected a reduction of feature-specific activity, when the meaning of the verbs is processed in a preceding context phase. Repetition of the conceptual information should lead to a reduced activity in the respective modality-specific system. The repetition of sound verbs, but not of action verbs, should result in a specific reduction of the activity within the auditory brain system. The repetition of action verbs, but not of sound verbs, in turn, should cause a reduction of activation in the motor system. As repetition of a given feature may reduce activity within corresponding modality-specific cortex below baseline, feature-specific ERP effects should not simply disappear, but are expected to exhibit a reversed polarity compared with Experiment 1 similar to earlier observations with nouns (Trumpp et al., 2014): Action verbs should elicit a relatively more negative potential compared with sound concepts at central and parietal electrodes.

### Materials and methods

#### Participants

Twenty-three (Age: 19–28 years, mean: 22.9 years, 12 female) right-handed volunteers (according to Oldfield, 1971), different from those of Experiment 1, participated in Experiment 2. Three subjects were excluded from analyses because of bilingualism, considerably long reaction times (RTs deviating 2 *SD*s from the sample mean.) or excessive artifacts in the EEG data. The final sample consisted of 20 data sets. Participants were native German speakers with normal or corrected to normal vision and no history of any psychiatric or neurological disorders.

#### Stimuli

The same 40 action and 40 sound verbs of Experiment 1 were used as critical stimuli for Experiment 2. For the context phase preceding the main experiment, each critical verb was paired with a semantically related context noun for the contextual matching condition according to four types of relations with descending priority. For sound verbs: (1) object that generates a sound (dog—to bark), (2) object which is manipulated and thereby elicits a sound (bell–to ring), (3) specific organ that elicits a sound (mouth—to speak), and (4) situation where sound occurs (cold—to sneeze). Context nouns for action verbs were chosen according to an equivalent pattern: (1) action object (trailer—to pull), (2) action instrument (chainsaw—to cut), (3) acting effector (leg—to run) or (4) situation (playground—to slide). Eighty different nouns were selected for contextual non-matching condition according to the criteria indicated above. For the contextual non-matching condition, 40 action and 40 sound verbs different from the critical verbs were chosen, which showed similar emotional, visual as well as familiarity ratings as the 80 critical verbs. Word length of the verbs of the non-matching condition was matched with the critical verbs of the matching condition as closely as possible. Context nouns of the matching and non-matching conditions were also equated to word length. The distribution of the different relations between verb and context noun were also kept comparable across all experimental conditions. Please note that all critical sound and action words used for the main experiment were presented in a contextual matching condition. Stimuli for the context experiment were tested in several pilot experiments with a total of 15 participants in order to achieve a comparable level of difficulty for action, sound, and control conditions. Participants were presented with context nouns followed by action or sound verbs, which could be semantically related (matching) or not related (non-matching) to the context. Participants were instructed to decide whether both words were semantically related or not (for the precise procedure of this context decision task, see above). Control verbs from Experiment 1 were not included because it was difficult to create a context condition with a performance level comparable to the critical action and sound words.

#### Procedure

Experiment 2 consisted of two sessions: A first session with a context decision task and a second one with a lexical decision task. In the context decision task, 40 critical action verbs and 40 sound verbs with 80 matching and 80 non-matching context-verb filler pairs were randomly presented to the participants. Words were shown in white font on a black background (16 points character height; viewing angle about 3° horizontally and 1° vertically). Each trial started with the presentation of a fixation cross for 500 ms, followed by the presentation of the context noun for 400 ms. After a clear screen for 500 ms, the target verb was presented for 500 ms. Participants were instructed to decide as fast and as accurately as possible whether the two words were semantically related. They were instructed to press a key with the index finger in response to related word pairs and a different key with the middle finger in response to unrelated word pairs. After the response another clear screen appeared, followed by a pause indicated by three hash marks which lasted 1500 ms on average.

The second task was a lexical decision task with sound and action verbs as in Experiment 1 except that control words and the matched pseudowords were discarded yielding 160 trials in total. Participants were informed that some of the verbs from the first task appeared again. Prior to each session, participants were able to practice the task with stimuli not used for the main experiments.

#### EEG recording, signal extraction, and data analysis

In the lexical decision task session, EEG data were recorded and analyzed as described in Experiment 1. The mean number of artifact-free EEG-segments of trials with correct responses did not significantly differ between sound (38.8; *SD* =.89) and action verbs (38.85; *SD* = 1.31) [*F*_(1, 19)_ = 0.03; *p* = 0.87]. Non-parametric cluster permutation tests with two conditions were calculated based on two-tailed paired *t*-tests and were otherwise comparable to the procedure of Experiment 1.

### Results

#### Behavioral results

Participants responded in the context decision task with a mean accuracy of 97% (*SD* = 2.14) and in the lexical decision task with a mean accuracy of 96% (*SD* = 3.13). Number of errors did neither differ between action (1.65) and sound verbs (2.05) in the context decision task [*F*_(1, 19)_ = 1.15; *p* = 0.30] nor in the lexical decision task (action verbs = 0.8, sound verbs = 0.85) [*F*_(1, 19)_ = 0.03; *p* = 0.86]. For RT analysis, mean RT of correct responses was calculated for each word category and experimental session. Outlying reaction times (mean +/– 2 *SD*) were rejected from analysis (context decision: 4.18%; lexical decision: 4.69%). Mean RT did not differ for critical action (653 ms) and sound verbs (639 ms) [*F*_(1, 19)_ = 1.54; *p* = 0.23] in the semantic decision task. In the lexical decision task, RT difference between sound and action words was small, but significant [action verbs: 562 ms, sound verbs: 575 ms, *F*_(1, 19)_ = 4.47; *p* = 0.048; $\eta_{p}^{2}$ = 0.19].

##### Conjoint analyses of RT and ER for Experiments 1 and 2

Conjoint analyses of RT data of Experiments 1 and 2 revealed no significant interactions between the factors feature type and experiment (*p* = 0.14). For error rates, however, a significant interaction between feature type and experiment was found [*F*_(1, 40)_ = 14.52; *p* = 0.0004; ηp^2^ = 0.27]. According to *post-hoc* tests error rate was higher for sound words in Experiment 1 compared to any other condition (*p* = 0.0002).

#### Electrophysiological results

**Figure 4:**
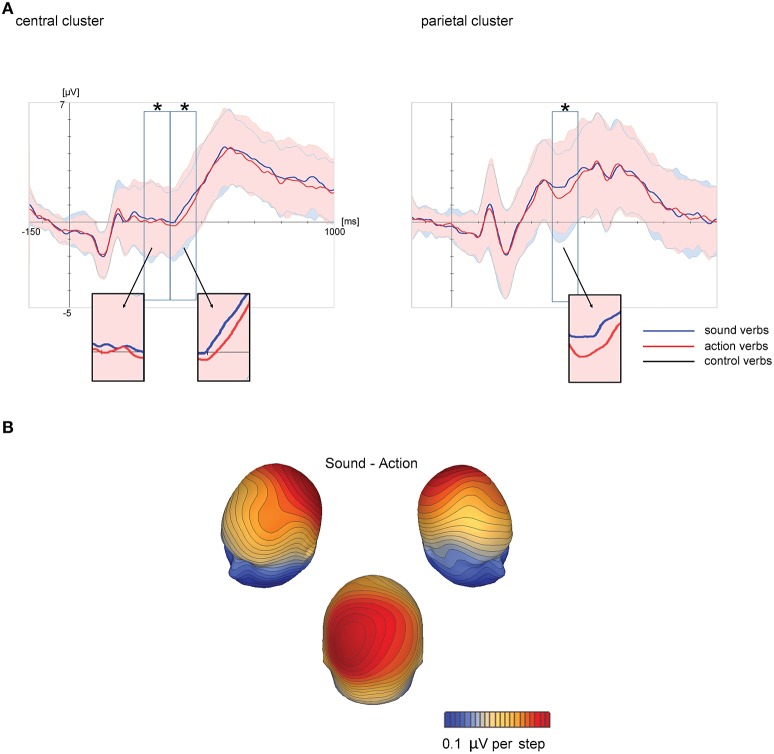
**Grand-averaged ERPs at central and parietal scalp regions, averaged across electrode sites within each scalp region in Experiment 2 (A)** as a function of feature type (action and sound verbs). In Experiment 2, the control verbs were omitted. Time windows analyzed statistically (180–280, 280–380, and 380–480 ms) are marked by black rectangles. Colored shadings indicate standard deviations. Significant ERP effects of feature type are highlighted by asterisks. Corresponding topographical potential maps at the time point of maximum global field power are shown for ERP differences between sound and action verbs are visualized **(B)**.

**Figure 5:**
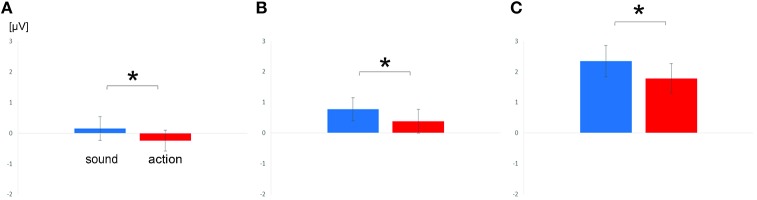
**Means and standard errors of analyzed scalp regions in Experiment 2**. Depicted are mean ERPs of sound and action over central scalp regions for the time window 280–380 ms **(A)** and 380–480 ms **(B)** and over parietal scalp regions for the time window 380–480 ms **(C)**. Significant effects are highlighted by asterisks.

Inspection of ERPs suggested an effect of feature type. In the time segments later than 280 ms, the effect of feature type was reversed compared with Experiment 1 with sound verbs eliciting more positive scalp potentials than action words in the central scalp region (Figure 4A). Analyzed time windows and scalp regions of the lexical decision task were the same as in Experiment 1. Means and standard errors of ERPs are depicted in Figure 5. As a direct comparison of ERP effects between both experiments, a conjoint analysis of Experiment 1 and 2 was additionally calculated.

##### Time window 180–280 ms

The first ANOVA including the factor scalp region yielded a significant main effect of scalp region [*F*_(1, 19)_ = 13.69; *p* = 0.002; $\eta_{p}^{2}$ = 0.42], but no interaction with feature type (*p* = 0.99. No significant main effect or interactions were found in the separate ANOVAs.

##### Time window 280–380 ms

The first ANOVA including the factor scalp region yielded a significant main effect of scalp region [*F*_(1, 19)_ = 34.60; *p* < 0.0001; $\eta_{p}^{2}$ = 0.65] and a significant main effect of feature type [*F*_(1, 19)_ = 7.25; *p* = 0.014; $\eta_{p}^{2}$ = 0.28], but no interaction of scalp region and feature type (*p* = 0.12). Separate ANOVAs in each scalp region only confirmed the effect for feature type in the central scalp region [*F*_(1, 19)_ = 8.74; *p* = 0.008; $\eta_{p}^{2}$ = 0.32]. Sound verbs were associated with relatively more positive scalp potentials than action verbs.

##### Time window 380–480 ms

The ANOVA including the factor scalp region revealed a main effect of scalp region [*F*_(1, 19)_ = 15.15; *p* = 0.001; $\eta_{p}^{2}$ = 0.44] and a main effect of feature type [*F*_(1, 19)_ = 19.17; *p* = 0.0003; $\eta_{p}^{2}$ = 0.50], but no interaction of scalp region and feature type [*p* = 0.37]. The separate ANOVAs in each scalp region confirmed main effects of feature type at central [*F*_(1, 19)_ = 6.83; *p* = 0.017; $\eta_{p}^{2}$ = 0.26] and parietal electrodes [*F*_(1, 19)_ = 15.85; *p* = 0.0008; $\eta_{p}^{2}$ = 0.45]. In both scalp regions, sound verbs elicited more positive scalp potentials than action verbs.

##### Control analyses with regard to response speed and errors

As in Experiment 1, we assessed whether feature-specific ERP effects would depend on overall response speed or error, by dividing the participants in high and low performing groups based on a median split. The ANOVAs with the between-subject factor response speed and error rate group, respectively, revealed no significant interactions with the factor feature type (all *p* > 0.06).

##### Non-parametric cluster permutation tests

Cluster permutation tests revealed a significant difference between sound and action verbs first within a short time window between 142 and 196 ms in a cluster of occipito-parietal electrodes and later within 370–486 ms in a cluster of centro-parietal electrodes (see Table 3, Figure 3B). Sound verbs elicited a more positive scalp potential than action verbs. The effect in the centro-parietal cluster was accompanied by a polarity-reversed effect in a fronto-temporal cluster (388–470 ms), as it is typical for a bipolar potential field in average-referenced data sets (see Figure 3).

**Table 3:** **Results of cluster permutation tests for Experiment 2**.

**Electrodes within cluster**	**Time window**	***p*-value**
TP9, P9, O9, O7, P7, TP7, PO3, PO1, O10, Iz, Oz	142–196 ms	0.001
O1, P7, P5, PO3, PO1, P1, CP3, C3, FC1, CP1, Cz, Oz, O2, P8, PO4, Pz, P2, CP2, Cpz	370–486 ms	<0.00001
FPz, AFz, F10, FT10, TP10, TP8, T8, FT8, AF8, FP2, AF4, F9, FC6, Nz	388–470 ms	0.002

##### Parametric conjoint analyses of Experiments 1 and 2

Conjoint analyses were only calculated for the central scalp region at the time window from 280 to 380 ms, where feature-dependent effects occurred both in Experiment 1 and 2. An ANOVA with the between-subject factor Experiment revealed a significant interaction between feature type and experiment [*F*_(1, 40)_ = 16.31; *p* = 0.0002; $\eta_{p}^{2}$ = 0.29]. This interaction was due to a polarity-reversal of the feature-specific ERP effects across experiments as already indicated by the analyses of each experiment.

### Discussion

In Experiment 2, we investigated action and sound verb processing, when critical stimuli were presented in a preceding context phase. Between about 300 and 500 ms, an ERP effect polarity-reversed compared with that found in Experiment 1 was obtained with more positive ERPs for sound than for action verbs. These ERP effects are in line with earlier demonstrations of a reduction of feature-specific ERP activity in noun processing as a function of priming, leading to a polarity-reversal of feature-specific effects compared to the unprimed condition (Kiefer, 2005; Sim and Kiefer, 2005; Trumpp et al., 2013b, 2014). Data-driven cluster-based permutation tests mainly confirmed the centro-parietal feature effect observed in the parametric analyses based on apriorily selected time windows and scalp regions of interest. There were only slight differences between parametric analysis and cluster permutation tests: Cluster permutation tests revealed an additional short-lived earlier occipto-parietal feature effect, and the time range of the later centro-parietal effect was somewhat narrower as in the parametric analyses. The comparable RT and ER pattern for all “yes” responses in the semantic decision task of experiment 2 indexes that semantic relatedness between verbs and context words was successfully matched for action and sound verbs. We can therefor exclude that the ERP effects of feature type reflect differential facilitation induced by the context words. The present results thus indicate that previous exposure of stimuli in a context phase modulates subsequent feature-specific verb processing similar to priming effects obtained with nouns. This suggests that action and sound features of verbs are processed in different neural circuits comparable to nouns.

## General discussion

In two ERP experiments, the present work investigated semantic processing of action and sound verbs and tested whether feature-specific ERP effects previously obtained with nouns (Trumpp et al., 2013b, 2014) can also be observed for verbs. In Experiment 1, ERPs of action and sound verbs showed a differential ERP polarity pattern in parietal and central scalp regions. The present ERP effects of action and sound verbs are largely comparable with earlier findings for action- and sound-related nouns: Similar to action nouns (Kiefer, 2001, 2005; Trumpp et al., 2013b, 2014), action verbs elicited a relatively more positive potential compared to control and sound verbs. Likewise, similar to sound nouns (Kiefer et al., 2008; Trumpp et al., 2013b, 2014), sound verbs elicited a relatively more negative scalp potential compared to control and action verbs. This feature-specific ERP effect emerged relatively early in Experiment 1 starting at 180 ms. These early ERP differences suggest that underlying processes reflect rapid access to conceptual word features (Kiefer et al., 2007), but do not depend on imagery or semantic elaboration processes (Machery, 2007; Chatterjee, 2010), which are known to modulate late ERP components (Nittono et al., 2002; Swaab et al., 2002; Barber et al., 2013).

Although there were slight differences in RTs and error rate for action and sound words within and between experiments, it is unlikely that they reflect systematic difficulty differences across verbs categories and experiments. For the RT data, the conjoint analysis did not yield significant differences between experiments. With regard to error rate, more errors were made for sound verbs compared with action verbs in Experiments 1 and 2 and with sound verbs in Experiment 2. Although the stimuli were the same, this increased error rate for sound verbs was only found in Experiment 1 and was not replicated in Experiment 2. Thus, this observation does not seem to indicate a specific difficulty to recognize sound verbs. In a similar vein, the mean number of EEG segments available for ERP analyses was slightly lower for sound verbs than for action and control verbs in Experiment 1, although the overall number of available segments was high and the difference was small. In order to assess how response speed or the likelihood to make an error affected the ERP effects of feature type, we performed control analyses comparing subgroups of participants with high- vs. low response speed or error rate. In these analyses, response speed had no significant effects at all. The factor error rate group (high. vs. low) moderated the ERP effect of feature type only for ERPs in the fronto-central scalp region of the time window 280–380 ms in Experiment 1. This interaction was due to a larger ERP difference between sound and action verbs for participants with a high error rate, although the same ERP differences were present in participants with a low error rate. It is thus very unlikely that our ERP effects were caused by an unspecific lexical decision difficulty or differences in signal-to-noise-ratio across conditions.

To ensure that possible effects may not refer to any psycholinguistic differences in the word material, we carefully matched several parameters within the word sets and amongst them. Nevertheless, there were small differences in familiarity between action and sound verbs that just failed to reach the selected significance level (*p* = 0.05). It should be noted that action verbs and control verbs were even more similar with regard to familiarity (*p* = 0.58). Although familiarity of action and control verbs was very comparable, ERPs to action verbs differed from ERPs to control verbs and not only from those to sound verbs (Experiment 1; in Experiment 2 control verbs were not presented). Furthermore, similar to sound verbs, control verbs elicited less positive ERPs compared with action verbs. As these ERP differences went in the same direction, it is unlikely that the present feature-specific ERP effects simply reflect differential familiarity.

The different polarity and topography of ERPs for action and sound verbs indicates that conceptual action and sound information is processed in different neural circuits (see Figures 1B, 4B). Although the localizational value of ERPs must be viewed with caution (Nunez, 1981), the topography of the present effects with verbs were largely compatible with earlier combined ERP and neuroimaging studies on action vs. sound processing in nouns: Processing of semantic action information was associated in the ERPs recordings with a fronto-central positive potential, while corresponding functional magnetic resonance imaging (fMRI) results indicated enhanced activity within frontal and parietal motor areas as well as in occipito-temporal motion-related areas (Hoenig et al., 2008). Processing semantic sound information, in contrast, was associated with more negative ERPs at fronto-central electrodes (Kiefer et al., 2008; Trumpp et al., 2013b, 2014), where acoustically evoked potentials are typically recorded (Näätänen, 1992). The accompanying fMRI results indicated activity within auditory association areas of posterior superior and middle temporal cortex (Kiefer et al., 2008). In the context of the relatively small ERP effects of the present study, it does not seem reasonable to perform source analyses due to the low signal-to noise-ratio. More reliable and detailed spatial information may be provided by neuroimaging techniques with higher spatial resolution in future studies, although they lack the high temporal resolution of ERPs. The present result pattern of ERP differences is nevertheless consistent with feature-specific neural processing of action and sound verbs, possibly in action-related and auditory brain areas.

Unlike for nouns (Kiefer, 2001, 2005; Hoenig et al., 2008; Trumpp et al., 2013b, 2014), however, the present ERPs to action verbs dissociated from those to sound verbs also at parietal electrodes. Furthermore, action verbs differed from control verbs with low action relevance only over the parietal scalp region. It must remain open whether this parietal feature-specific ERP effect in action verbs reflects a differential activation of the frontal and parietal motor system compared with action nouns. This difference across studies could be accounted for by several factors such as different tasks (lexical decision in the present experiment, various tasks in the other studies such as categorization, property verification or silent reading) and different comparison conditions (visual concepts, control concepts with low action relevance), in addition to word class differences. In order to keep the duration of the experiments reasonable, we refrained from including action and sound nouns within the present experiments.

It was not possible to completely match the visual content of action and sound verbs, although the matching of action verbs to the control verbs was nearly perfect. As actions are typically related to visual properties of an object or a situation (Tyler and Moss, 2001), action verbs were predominantly highly rated in visual content. This rendered it difficult to separate this linkage without having to rely on very atypical word material. As visuomotor features of an object are represented in parietal cortical areas (Jeannerod et al., 1995; Jeannerod, 2001; Vogt et al., 2003), the combination of action and visual features in the present verbs could therefore account of the ERP effects at parietal electrodes, which were not observed in nouns. Further confounding factors which could make it difficult to compare ERP effects of verbs and nouns derive from a more complex processing of verbs compared to nouns (Ehrlich and Rayner, 1981). This may also may explain why the present ERP feature-specific effects obtained with verbs partially differ from those obtained earlier with nouns with regard to precise topography and onset, which was 100 ms earlier in nouns.

In Experiment 2, a context decision task was implemented prior to the lexical decision task. While keeping visual stimulation and lexical task equal to Experiment 1, we were able to assess how pre-activation of action and sound concepts associated with verbs in the preceding context phase affects feature-specific ERP effects. In time windows from between 280 and 480 ms, feature-specific ERP effects exhibited in Experiment 2 a reversed polarity compared with Experiment 1: Sound verbs elicited more positive ERPs than action verbs in the central scalp region. These feature-related differences must arise from differentially activated semantic representations because other factors such as responses or decision were held constant across verb categories. This reversed polarity pattern for primed sound and action features have already been found in earlier studies with nouns (e.g., Trumpp et al., 2013b, 2014). Pre-activation of the verbs in the context phase resulted in a modulation of feature-specific activity, presumably due to a specific deactivation (Grill-Spector et al., 2006; Horner and Henson, 2008) of the motor or auditory brain areas below baseline, which process the corresponding conceptual verb feature. Such deactivations are supposed to lead to a reduction of feature-specific ERP activity, i.e., the negative potential shift for sound verbs and the positive potential shift for action verbs as observed in Experiment 1, resulting in relatively more positive potentials for sound verbs and relatively more negative potentials for action verbs, rather than null effecs. Conjoint analyses of Experiment 1 and 2 showed that these differential ERP effects across experiments were statistically reliable.

The results of both experiments implicate a distinct conceptual feature-dependent processing of verbs in line with a grounding of conceptual representations in action and perception. According to grounded cognition theories (Gallese and Lakoff, 2005; Martin, 2007; Barsalou, 2008; Pulvermüller and Fadiga, 2010; Meteyard et al., 2012; Kiefer and Barsalou, 2013) concepts are, depending on individual experience, represented in at least partially different sensory and/or motor areas that are activated during action and/or perception. In contrast, the present findings are difficult to reconcile with amodal theories of conceptual represention (Collins and Loftus, 1975; Pylyshyn, 1980; Anderson, 1983; Mahon and Caramazza, 2009), which propose conceptual processing of verbs irrespective of the feature type. However, the present results do not preclude the possible existence of a supramodal convergence zone or semantic hub, in addition to modality-specific semantic systems, as proposed by hybrid grounded cognition approaches (Louwerse and Jeuniaux, 2008; Kiefer and Pulvermüller, 2012; Mirabella et al., 2012; Arbib et al., 2014; García and Ibáñez, 2016a,b).

Our study extends previous work by suggesting that verbs are not only linked to action, the main focus of previous studies (Pulvermüller et al., 1999a,b; Vinson and Vigliocco, 2002; Bedny et al., 2008; Boulenger et al., 2008; Bedny and Caramazza, 2011), but are also grounded in the auditory system. We propose that similar to nouns the semantic content of verbs is established by a differential contribution of sensory and motor features, presumably depending on the perception and action experience during concept acquisition. The heterogeneous modality-specific semantic content of verbs might explain, why the processing of verbs have not been consistently found to involve the motor system (Damasio and Tranel, 1993; Perani et al., 1999; Vigliocco et al., 2006). Of course, the present observation of differential feature-specific brain activity only provides correlational information, but does not indicate, whether motor or sound information is necessary to understand the meaning of verbs (Mahon and Caramazza, 2008; Caramazza et al., 2014). The functional relevance of the sensorimotor systems in conceptual processing of verbs needs to be demonstrated in future studies involving TMS, brain-damaged patients or behavioral interference paradigms. Since we only included verbs with concrete semantic content, it must remain open whether and how the meaning of abstract verbs such as “to think” is grounded in modality-specific systems. Presumably, in addition to perception and action, brain systems involved in introspection and emotion are important (Vigliocco et al., 2009; Kiefer and Barsalou, 2013).

In conclusion, our ERP study provides evidence for a differential processing of action and sound verbs in congruency with previous findings on the sensory and motor foundations of the meaning of concrete nouns. The results indicate that the meaning of verbs is not only linked to the motor brain system, but also to the auditory system depending on conceptual feature relevance. In congruency with grounded cognition theories, we propose that, similar to concrete nouns, the meaning of verbs is established by differential composition of features represented in modality-specific brain systems.

## Ethics statement

The procedures of this study have been proved by the Ethics Committee of Ulm University. Participants received written information about the experimental procedure and signed a written informed consent.

## Author contributions

MP, NT, and MK planned the study design; NT and MK supervised the study; MP performed data acquisition, analyzed the data, and wrote the first draft of the paper. MP, MK, and NT revised the manuscript. All authors approved the final version of the manuscript.

### Conflict of interest statement

The authors declare that the research was conducted in the absence of any commercial or financial relationships that could be construed as a potential conflict of interest.

## References

[B1] Anderson A. J. Bruni E. Lopopolo A. Poesio M. Baroni M. (2015). Reading visually embodied meaning from the brain: visually grounded computational models decode visual-object mental imagery induced by written text. Neuroimage 120, 309–322. 10.1016/j.neuroimage.2015.06.093 26188260

[B2] Anderson J. R. (1983). The Architecture of Cognition. Hillsdayle, NJ: Lawrence Erlbaum Associates, Inc.

[B3] Arbib M. A. Gasser B. Barrès V. (2014). Language is handy but is it embodied? Neuropsychologia 55, 57–70. 10.1016/j.neuropsychologia.2013.11.004 24252354

[B4] Arévalo A. L. Baldo J. V. Dronkers N. F. (2012). What do brain lesions tell us about theories of embodied semantics and the human mirror neuron system? Cortex 48, 242–254. 10.1016/j.cortex.2010.06.001 20621292 PMC3615255

[B5] Arévalo A. Perani D. Cappa S. F. Butler A. Bates E. Dronkers N. (2007). Action and object processing in aphasia: from nouns and verbs to the effect of manipulability. Brain Lang. 100, 79–94. 10.1016/j.bandl.2006.06.012 16949143

[B6] Baayen R. H. Piepenbrock R. Gulikers L. (1995). The CELEX Lexical Database (Release 2) [CD-ROM]. Philadelphia, PA: Linguistic Data Consortium; University of Pennsylvania [Distributor].

[B7] Bak T. H. (2013). The neuroscience of action semantics in neurodegenerative brain diseases. Curr. Opin. Neurol. 26, 671–677. 10.1097/WCO.0000000000000039 24184973

[B8] Barber H. A. Kousta S. T. Otten L. J. Vigliocco G. (2010). Event-related potentials to event-related words: grammatical class and semantic attributes in the representation of knowledge. Brain Res. 1332, 65–74. 10.1016/j.brainres.2010.03.014 20230804

[B9] Barber H. A. Otten L. J. Kousta S. T. Vigliocco G. (2013). Concreteness in word processing: ERP and behavioral effects in a lexical decision task. Brain Lang. 125, 47–53. 10.1016/j.bandl.2013.01.005 23454073

[B10] Barsalou L. W. (2008). Grounded cognition. Annu. Rev. Psychol. 59, 617–645. 10.1146/annurev.psych.59.103006.093639 17705682

[B11] Barsalou L. W. Kyle Simmons W. Barbey A. K. Wilson C. D. (2003). Grounding conceptual knowledge in modality-specific systems. Trends Cogn. Sci. 7, 84–91. 10.1016/S1364-6613(02)00029-3 12584027

[B12] Bedny M. Caramazza A. (2011). Perception, action, and word meanings in the human brain: the case from action verbs. Ann. N. Y. Acad. Sci. 1224, 81–95. 10.1111/j.1749-6632.2011.06013.x 21486297

[B13] Bedny M. Caramazza A. Grossman E. Pascual-Leone A. Saxe R. (2008). Concepts are more than percepts: the case of action verbs. J. Neurosci. 28, 11347–11353. 10.1523/JNEUROSCI.3039-08.2008 18971476 PMC2752357

[B14] Bertrand O. Perrin F. Pernier J. (1985). A theoretical justification of the average reference in topographic evoked potential studies. Electroencephalogr. Clin. Neurophysiol. 62, 462–464. 10.1016/0168-5597(85)90058-9 2415344

[B15] Bierwisch M. Schreuder R. (1992). From concepts to lexical items. Cognition 42, 23–60. 10.1016/0010-0277(92)90039-K 1582158

[B16] Bird H. Franklin S. Howard D. (2001). Age of acquisition and imageability ratings for a large set of words, including verbs and function words. Behav. Res. Methods Instrum. Comput. 33, 73–79. 10.3758/BF03195349 11296722

[B17] Bocanegra Y. García A. M. Pineda D. Buriticá O. Villegas A. Lopera F.. (2015). Syntax, action verbs, action semantics, and object semantics in Parkinson's disease: dissociability, progression, and executive influences. Cortex 69, 237–254. 10.1016/j.cortex.2015.05.022 26103601

[B18] Boulenger V. Mechtouff L. Thobois S. Broussolle E. Jeannerod M. Nazir T. A. (2008). Word processing in Parkinson's disease is impaired for action verbs but not for concrete nouns. Neuropsychologia 46, 743–756. 10.1016/j.neuropsychologia.2007.10.007 18037143

[B19] Capitani E. Laiacona M. Mahon B. Caramazza A. (2003). What are the facts of semantic category-specific deficits? A critical review of the clinical evidence. Cogn. Neuropsychol. 20, 213–261. 10.1080/02643290244000266 20957571

[B20] Caramazza A. Anzellotti S. Strnad L. Lingnau A. (2014). Embodied cognition and mirror neurons: a critical assessment. Annu. Rev. Neurosci. 37, 1–15. 10.1146/annurev-neuro-071013-013950 25032490

[B21] Cardona J. F. Gershanik O. Gelormini-Lezama C. Houck A. L. Cardona S. Kargieman L.. (2013). Action-verb processing in Parkinson's disease: new pathways for motor-language coupling. Brain Struct. Funct. 218, 1355–1373. 10.1007/s00429-013-0510-1 23412746

[B22] Chao L. L. Haxby J. V. Martin A. (1999). Attribute-based neural substrates in temporal cortex for perceiving and knowing about objects. Nat. Neurosci. 2, 913–919. 10.1038/13217 10491613

[B23] Chao L. L. Martin A. (1999). Cortical regions associated with perceiving, naming and knowing about colors. J. Cogn. Neurosci. 11, 25–35. 10.1162/089892999563229 9950712

[B24] Chao L. L. Martin A. (2000). Representation of manipulable man-made objects in the dorsal stream. Neuroimage 12, 478–484. 10.1006/nimg.2000.0635 10988041

[B25] Chatterjee A. (2010). Disembodying cognition. Lang. Cogn. 2, 79–116. 10.1515/langcog.2010.004 20802833 PMC2927131

[B26] Collins A. M. Loftus E. F. (1975). A spreading–activation theory of semantic processing. Psychol. Rev. 82, 407–428. 10.1037/0033-295X.82.6.407

[B27] Colombo L. Burani C. (2002). The influence of age of acquisition, root frequency, and context availability in processing nouns and verbs. Brain Lang. 81, 398–411. 10.1006/brln.2001.2533 12081408

[B28] Damasio A. R. Tranel D. (1993). Nouns and verbs are retrieved with differently distributed neural systems. Proc. Natl. Acad. Sci. U.S.A. 90, 4957–4960. 10.1073/pnas.90.11.4957 8506341 PMC46632

[B29] Daniele A. Giustolisi L. Silveri M. C. Colosimo C. Gainotti G. (1994). Evidence for a possible neuroanatomical basis for lexical processing of nouns and verbs. Neuropsychologia 32, 1325–1341. 10.1016/0028-3932(94)00066-2 7533275

[B30] de Zubicaray G. I. Wilson S. J. Mcmahon K. L. Muthiah S. (2001). The semantic interference effect in the picture-word paradigm: An event-related fMRI study employing overt responses. Hum. Brain Mapp. 14, 218–227. 10.1002/hbm.1054 11668653 PMC6871995

[B31] Devereux B. J. Clarke A. Marouchos A. Tyler L. K. (2013). Representational similarity analysis reveals commonalities and differences in the semantic processing of words and objects. J. Neurosci. 33, 18906–18916. 10.1523/JNEUROSCI.3809-13.2013 24285896 PMC3852350

[B32] Dijkstra K. Post L. (2015). Mechanisms of embodiment. Front. Psychol. 6:1525. 10.3389/fpsyg.2015.01525 26528203 PMC4606013

[B33] Ehrlich S. F. Rayner K. (1981). Contextual effects on word perception and eye-movements during reading. J. Verbal Learn. Verbal Behav. 20, 641–655. 10.1016/S0022-5371(81)90220-6

[B34] Fairhall S. L. Caramazza A. (2013). Brain regions that represent amodal conceptual knowledge. J. Neurosci. 33, 10552–10558. 10.1523/JNEUROSCI.0051-13.2013 23785167 PMC6618586

[B35] Gallese V. Lakoff G. (2005). The brain's concepts: the role of the sensory-motor system in conceptual knowledge. Cogn. Neuropsychol. 22, 455–479. 10.1080/02643290442000310 21038261

[B36] García A. M. Ibáñez A. (2014). Words in motion: motor-language coupling in Parkinson's disease. Transl. Neurosci. 5, 152–159. 10.2478/s13380-014-0218-6

[B37] García A. M. Ibáñez A. (2016a). Hands typing what hands do: action-semantic integration dynamics throughout written verb production. Cognition 149, 56–66. 10.1016/j.cognition.2016.01.011 26803393

[B38] García A. M. Ibáñez A. (2016b). A touch with words: dynamic synergies between manual actions and language. Neurosci. Biobehav. Rev. 68, 59–95. 10.1016/j.neubiorev.2016.04.022 27189784

[B39] Gentner D. (1982). Why nouns are learned before verbs: linguistic relativity versus natural partitioning, in Language Development, Vol. 2, ed Kuczaj S. (Hillsdale, MI: Erlbaum), 301–333

[B40] Glenberg A. M. Gallese V. (2012). Action-based language: a theory of language acquisition, comprehension, and production. Cortex 48, 905–922. 10.1016/j.cortex.2011.04.010 21601842

[B41] Gold B. T. Balota D. A. Jones S. J. Powell D. K. Smith C. D. Andersen A. H. (2006). Dissociation of automatic and strategic lexical-semantics: functional magnetic resonance imaging evidence for differing roles of multiple frontotemporal regions. J. Neurosci. 26, 6523–6532. 10.1523/JNEUROSCI.0808-06.2006 16775140 PMC6674026

[B42] Grill-Spector K. Henson R. Martin A. (2006). Repetition and the brain: neural models of stimulus-specific effects. Trends Cogn. Sci. 10, 14–23. 10.1016/j.tics.2005.11.006 16321563

[B43] Hauk O. Johnsrude I. Pulvermüller F. (2004). Somatotopic representation of action words in human motor and premotor cortex. Neuron 41, 301–307. 10.1016/S0896-6273(03)00838-9 14741110

[B44] Heister J. Wurzner K. M. Bubenzer J. Pohl E. Hanneforth T. Geyken A. (2011). dlexDB - A lexical database for the psychological and linguistic research. Psychol. Rundsch. 62, 10–20. 10.1026/0033-3042/a000029

[B45] Henson R. N. (2003). Neuroimaging studies of priming. Prog. Neurobiol. 70, 53–81. 10.1016/S0301-0082(03)00086-8 12927334

[B46] Hoenig K. Sim E.-J. Bochev V. Herrnberger B. Kiefer M. (2008). Conceptual flexibility in the human brain: dynamic recruitment of semantic maps from visual, motion and motor-related areas. J. Cogn. Neurosci. 20, 1799–1814. 10.1162/jocn.2008.20123 18370598

[B47] Horner A. J. Henson R. N. (2008). Priming, response learning and repetition suppression. Neuropsychologia 46, 1979–1991. 10.1016/j.neuropsychologia.2008.01.018 18328508 PMC2430995

[B48] Humphreys G. W. Riddoch M. J. Quinlan P. T. (1988). Cascade processes in picture identification. Cogn. Neuropsychol. 5, 67–103. 10.1080/02643298808252927

[B49] Jeannerod M. (2001). Neural simulation of action: a unifying mechanism for motor cognition. Neuroimage 14, S103–S109. 10.1006/nimg.2001.0832 11373140

[B50] Jeannerod M. Arbib M. A. Rizzolatti G. Sakata H. (1995). Grasping objects: the cortical mechanisms of visuomotor transformation. Trends Neurosci. 18, 314–320. 10.1016/0166-2236(95)93921-J 7571012

[B51] Johansson R. S. Westling G. Bäckström A. Flanagan J. R. (2001). Eye-hand coordination in object manipulation. J. Neurosci. 21, 6917–6932. 11517279 10.1523/JNEUROSCI.21-17-06917.2001 PMC6763066

[B52] Jones S. S. Smith L. B. (1993). The Place of perception in children's concepts. Cogn. Dev. 8, 113–139. 10.1016/0885-2014(93)90008-S

[B53] Kauschke C. Stenneken P. (2008). Differences in noun and verb processing in lexical decision cannot be attributed to word form and morphological complexity alone. J. Psycholinguist. Res. 37, 443–452. 10.1007/s10936-008-9073-3 18452060

[B54] Kiefer M. (2001). Perceptual and semantic sources of category-specific effects in object categorization: event-related potentials during picture and word categorization. Mem. Cogn. 29, 100–116. 10.3758/BF03195745 11277454

[B55] Kiefer M. (2005). Repetition priming modulates category-related effects on event-related potentials: further evidence for multiple cortical semantic systems. J. Cogn. Neurosci. 17, 199–211. 10.1162/0898929053124938 15811233

[B56] Kiefer M. Barsalou L. W. (2013). Grounding the human conceptual system in perception, action, and internal states, in Action Science: Foundations of an Emerging Discipline, eds Prinz W. Beisert M. Herwig A. (Cambridge: MIT Press), 381–407.

[B57] Kiefer M. Pulvermüller F. (2012). Conceptual representations in mind and brain: theoretical developments, current evidence and future directions. Cortex 48, 805–825. 10.1016/j.cortex.2011.04.006 21621764

[B58] Kiefer M. Sim E.-J. Herrnberger B. Grothe J. Hoenig K. (2008). The sound of concepts: four markers for a link between auditory and conceptual brain systems. J. Neurosci. 28, 12224–12230. 10.1523/JNEUROSCI.3579-08.2008 19020016 PMC6671691

[B59] Kiefer M. Sim E.-J. Liebich S. Hauk O. Tanaka J. (2007). Experience-dependent plasticity of conceptual representations in human sensory-motor areas. J. Cogn. Neurosci. 19, 525–542. 10.1162/jocn.2007.19.3.525 17335399

[B60] Kiefer M. Trumpp N. Herrnberger B. Sim E.-J. Hoenig K. Pulvermüller F. (2012). Dissociating the representation of action- and sound-related concepts in middle temporal cortex. Brain Lang. 122, 120–125. 10.1016/j.bandl.2012.05.007 22726721

[B61] Kiefer M. Weisbrod M. Spitzer M. (1998). Zur funktionellen Neuroanatomie und Psychopathologie des semantischen Gedächtnisses. Psychol. Rundsch. 49, 132–143.

[B62] Levelt W. J. M. (1989). Speaking: From Intention to Articulation. Cambridge: MIT Press.

[B63] Louwerse M. Jeuniaux P. (2008). Language comprehension is both embodied and symbolic, in Symbols and Embodiment: Debates on Meaning and Cognition, eds Vega M. D. Glenberg A. Graesser A. (Oxford: Oxford University Press), 309–326.

[B64] Machery E. (2007). Concept empiricism: a methodological critique. Cognition 104, 19–46. 10.1016/j.cognition.2006.05.002 16814274

[B65] Mahon B. Z. Caramazza A. (2008). A critical look at the embodied cognition hypothesis and a new proposal for grounding conceptual content. J. Physiol. 102, 59–70. 10.1016/j.jphysparis.2008.03.004 18448316

[B66] Mahon B. Z. Caramazza A. (2009). Concepts and categories: a cognitive neuropsychological perspective. Annu. Rev. Psychol. 60, 27–51. 10.1146/annurev.psych.60.110707.163532 18767921 PMC2908258

[B67] Makeig S. Bell A. J. Jung T.-P. Ghahremani D. Sejnowski T. J. (1997). Blind separation of auditory event-related brain responses into independent components. Proc. Natl. Acad. Sci. U.S.A. 94, 10979–10984. 10.1073/pnas.94.20.10979 9380745 PMC23551

[B68] Maris E. Oostenveld R. (2007). Nonparametric statistical testing of EEG- and MEG-data. J. Neurosci. Methods 164, 177–190. 10.1016/j.jneumeth.2007.03.024 17517438

[B69] Martin A. (2007). The representation of object concepts in the brain. Annu. Rev. Psychol. 58, 25–45. 10.1146/annurev.psych.57.102904.190143 16968210

[B70] Meteyard L. Cuadrado S. R. Bahrami B. Vigliocco G. (2012). Coming of age: a review of embodiment and the neuroscience of semantics. Cortex 48, 788–804. 10.1016/j.cortex.2010.11.002 21163473

[B71] Mirabella G. Iaconelli S. Spadacenta S. Federico P. Gallese V. (2012). Processing of hand-related verbs specifically affects the planning and execution of arm reaching movements. PLoS ONE 7:e35403. 10.1371/journal.pone.0035403 22536380 PMC3335064

[B72] Moseley R. L. Pulvermüller F. (2014). Nouns, verbs, objects, actions, and abstractions: local fMRI activity indexes semantics, not lexical categories. Brain Lang. 132, 28–42. 10.1016/j.bandl.2014.03.001 24727103 PMC4029073

[B73] Moseley R. L. Kiefer M. Pulvermüller F. (2016). Grounding and embodiment of concepts and meaning: a neurobiological perspective, in Foundations of Embodied Cognition, eds Coello Y. Fischer M. H. (Oxon: Routledge), 93–113.

[B74] Näätänen R. (1992). Attention and Brain Function. Hillsdale, NJ: Erlbaum.

[B75] Nittono H. Suehiro M. Hori T. (2002). Word imageability and N400 in an incidental memory paradigm. Intern. J. Psychophys. 44, 219–229. 10.1016/S0167-8760(02)00002-8 12031296

[B76] Nunez P. L. (1981). Electrical Fields of the Brain: The Neurophysics of EEG. New York, NY: Oxford University Press.

[B77] Oldfield R. (1971). The assessment and analysis of handedness: the Edinburgh Inventory. Neuropsychologia 9, 97–113. 10.1016/0028-3932(71)90067-4 5146491

[B78] Palazova M. Mantwill K. Sommer W. Schacht A. (2011). Are effects of emotion in single words non-lexical? Evidence from event-related brain potentials. Neuropsychologia 49, 2766–2775. 10.1016/j.neuropsychologia.2011.06.005 21684295

[B79] Paradis M. (2004). A Neurolinguistic Theory of Bilingualism, Vol. 18 Amsterdam: John Benjamins Publishing.

[B80] Patterson K. Nestor P. J. Rogers T. T. (2007). Where do you know what you know? The representation of semantic knowledge in the human brain. Nat. Rev. Neurosci. 8, 976–987. 10.1038/nrn2277 18026167

[B81] Pechmann T. Garrett M. Zerbst D. (2004). The time course of recovery for grammatical category information during lexical processing for syntactic construction. J. Exp. Psychol. Learn. Mem. Cogn. 30, 723–728. 10.1037/0278-7393.30.3.723 15099139

[B82] Perani D. Cappa S. F. Schnur T. Tettamanti M. Collina S. Rosa M. M.. (1999). The neural correlates of verb and noun processing. A PET study. Brain 122, 2337–2344. 10.1093/brain/122.12.2337 10581226

[B83] Postle N. Mcmahon K. L. Ashton R. Meredith M. de Zubicaray G. I. (2008). Action word meaning representations in cytoarchitectonically defined primary and premotor cortices. Neuroimage 43, 634–644. 10.1016/j.neuroimage.2008.08.006 18786644

[B84] Preissl H. Pulvermüller F. Lutzenberger W. Birbaumer N. (1995). Evoked potentials distinguish between nouns and verbs. Neurosci. Lett. 197, 81–83. 10.1016/0304-3940(95)11892-Z 8545063

[B85] Pulvermüller F. (1999). Words in the brain's language. Behav. Brain Sci. 22, 253–336. 10.1017/S0140525X9900182X 11301524

[B86] Pulvermüller F. Fadiga L. (2010). Active perception: sensorimotor circuits as a cortical basis for language. Nat. Rev. Neurosci. 11, 351–360. 10.1038/nrn2811 20383203

[B87] Pulvermüller F. Hauk O. Nikulin V. V. Ilmoniemi R. J. (2005). Functional links between motor and language systems. Eur. J. Neurosci. 21, 793–797. 10.1111/j.1460-9568.2005.03900.x 15733097

[B88] Pulvermüller F. Lutzenberger W. Preissl H. (1999a). Nouns and verbs in the intact brain: evidence from event-related potentials and high-frequency cortical responses. Cereb. Cortex 9, 497–506. 10.1093/cercor/9.5.497 10450894

[B89] Pulvermüller F. Mohr B. Schleichert H. (1999b). Semantic or lexico-syntactic factors: what determines word-class specific activity in the human brain? Neurosci. Lett. 275, 81–84. 10.1016/S0304-3940(99)00724-7 10568504

[B90] Pylyshyn Z. W. (1980). Computation and cognition: issues in the foundation of cognitive science. Behav. Brain Sci. 3, 111–132. 10.1017/S0140525X00002053

[B91] Schomers M. R. Kirilina E. Weigand A. Bajbouj M. Pulvermüller F. (2015). Causal influence of articulatory motor cortex on comprehending single spoken words: TMS evidence. Cereb. Cortex 25, 3894–3902. 10.1093/cercor/bhu274 25452575 PMC4585521

[B92] Setti A. Caramelli N. Borghi A. M. (2009). Conceptual information about size of objects in nouns. Eur. J. Cogn. Psychol. 21, 1022–1044. 10.1080/09541440802469499

[B93] Shapiro K. Caramazza A. (2003). The representation of grammatical categories in the brain. Trends Cogn. Sci. 7, 201–206. 10.1016/S1364-6613(03)00060-3 12757821

[B94] Shapiro K. A. Moo L. R. Caramazza A. (2006). Cortical signatures of noun and verb production. Proc. Natl. Acad. Sci. U.S.A. 103, 1644–1649. 10.1073/pnas.0504142103 16432232 PMC1360518

[B95] Shapiro L. P. Zurif E. B. Grimshaw J. (1989). Verb processing during sentence comprehension: contextual impenetrability. J. Psycholinguist. Res. 18, 223–243. 2738859 10.1007/BF01067783

[B96] Shebani Z. Pulvermüller F. (2013). Moving the hands and feet specifically impairs working memory for arm- and leg-related action words. Cortex 49, 222–231. 10.1016/j.cortex.2011.10.005 22113187

[B97] Sim E.-J. Kiefer M. (2005). Category-related brain activity to natural categories is associated with the retrieval of visual features: evidence from repetition effects during visual and functional judgments. Cogn. Brain Res. 24, 260–273. 10.1016/j.cogbrainres.2005.02.006 15993764

[B98] Swaab T. Y. Baynes K. Knight R. T. (2002). Separable effects of priming and imageability on word processing: an ERP study. Cogn. Brain Res. 15, 99–103. 10.1016/S0926-6410(02)00219-7 12433385

[B99] Trumpp N. M. Kliese D. Hoenig K. Haarmaier T. Kiefer M. (2013a). Losing the sound of concepts: damage to auditory association cortex impairs the processing of sound-related concepts. Cortex 49, 474–486. 10.1016/j.cortex.2012.02.002 22405961

[B100] Trumpp N. M. Traub F. Kiefer M. (2013b). Masked priming of conceptual features reveals differential brain activation during unconscious access to conceptual action and sound information. PLoS ONE 8:e65910. 10.61371/journal.pone.0065910 23741518 PMC3669239

[B101] Trumpp N. M. Traub F. Pulvermuller F. Kiefer M. (2014). Unconscious automatic brain activation of acoustic and action-related conceptual features during masked repetition priming. J. Cogn. Neurosci. 26, 352–364. 10.1162/jocn_a_00473 24001008

[B102] Tulving E. (1972). Episodic and semantic memory, in Organization of Memory, eds Tulving E. Donaldson W. (New York, NY: Academic Press), 381–403.

[B103] Tyler L. K. Moss H. E. (2001). Towards a distributed account of conceptual knowledge. Trends Cogn. Sci. 5, 244–252. 10.1016/S1364-6613(00)01651-X 11390295

[B104] Vigliocco G. Meteyard L. Andrews M. Kousta S. (2009). Toward a theory of semantic representation. Lang. Cogn. 1, 219–247. 10.1515/LANGCOG.2009.011

[B105] Vigliocco G. Vinson D. P. Druks J. Barber H. Cappa S. F. (2011). Nouns and verbs in the brain: a review of behavioural, electrophysiological, neuropsychological and imaging studies. Neurosci. Biobehav. Rev. 35, 407–426. 10.1016/j.neubiorev.2010.04.007 20451552

[B106] Vigliocco G. Vinson D. P. Lewis W. Garrett M. F. (2004). Representing the meanings of object and action words: the featural and unitary semantic space hypothesis. Cogn. Psychol. 48, 422–488. 10.1016/j.cogpsych.2003.09.001 15099798

[B107] Vigliocco G. Warren J. Siri S. Arciuli J. Scott S. Wise R. (2006). The role of semantics and grammatical class in the neural representation of words. Cereb. Cortex 16, 1790–1796. 10.1093/cercor/bhj115 16421329

[B108] Vinson D. P. Vigliocco G. (2002). A semantic analysis of grammatical class impairments: semantic representations of object nouns, action nouns and action verbs. J. Neurolinguist. 15, 317–351. 10.1016/S0911-6044(01)00037-9

[B109] Visser M. Jefferies E. Lambon Ralph M. A. (2010). Semantic processing in the anterior temporal lobes: a meta-analysis of the functional neuroimaging literature. J. Cogn. Neurosci. 22, 1083–1094. 10.1162/jocn.2009.21309 19583477

[B110] Vogt S. Taylor P. Hopkins B. (2003). Visuomotor priming by pictures of hand postures: perspective matters. Neuropsychologia 41, 941–951. 10.1016/S0028-3932(02)00319-6 12667530

[B111] Zola D. (1984). Redundancy and word perception during reading. Percept. Psychophys. 36, 277–284. 10.3758/BF03206369 6522220

